# Selenium Supranutrition: Are the Potential Benefits of Chemoprevention Outweighed by the Promotion of Diabetes and Insulin Resistance?

**DOI:** 10.3390/nu5041349

**Published:** 2013-04-19

**Authors:** Caroline R. B. Rocourt, Wen-Hsing Cheng

**Affiliations:** Department of Nutrition and Food Science, University of Maryland, College Park, MD 20742, USA; E-Mail: crocourt@umd.edu

**Keywords:** selenium, supranutrition, type 2 diabetes, cancer

## Abstract

Selenium was considered a toxin until 1957, when this mineral was shown to be essential in the prevention of necrotic liver damage in rats. The hypothesis of selenium chemoprevention is principally formulated by the observations that cancer incidence is inversely associated with selenium status. However, recent clinical and epidemiological studies demonstrate a role for some selenoproteins in exacerbating or promoting other disease states, specifically type 2 diabetes, although other data support a role of selenium in stimulating insulin sensitivity. Therefore, it is clear that our understanding in the role of selenium in glucose metabolism and chemoprevention is inadequate and incomplete. Research exploring the role of selenium in individual healthcare is of upmost importance and possibly will help explain how selenium is a double-edged sword in the pathologies of chronic diseases.

## 1. Introduction

Selenium is an essential micronutrient found in Brazil nuts, chicken, fish, turkey, crab, nuts, cereal and eggs. Both selenium toxicity and deficiency primarily result from changes in dietary intake [1] or genetic variations [2,3]. The Food and Nutrition Board at the Institute of Medicine set the recommended daily allowance (RDA) of selenium at 55 µg/day for both adult males and females. This amount is based on the amount of dietary selenium needed to maximize the activity of the selenium-containing enzyme glutathione peroxidase in the plasma [4]. Dietary selenium can be incorporated into selenoproteins, which contain the amino acid, selenocysteine (Sec). A study by Kryukov *et al.* found that there are 25 selenoproteins in humans by identifying Sec insertion sequences (SECIS) elements in the genome [5]. SECIS elements are stem-loop structures in the 3’-untranslated regions of selenoprotein mRNA and facilitate the insertion of Sec into the nascent polypeptide chain in response to the UGA codon instead of signaling the termination of protein synthesis. The function of selenoproteins include acting in an antioxidant and anti-inflammatory [6] capacity and in immune [7] and thyroid [8] functions (for details, see [9]). 

The intake of selenium is highly varied among individuals and populations, because the selenium content of food varies widely depending on geographic locations and the type of food consumed [10,11]. For example, populations that live within a wide geographical belt that runs from northeast into southwest China, where the soil is selenium deficient, have an increased incidence of the Keshan disease, which is congestive cardiomyopathy seen in people who have a low body selenium status. Worldwide serum selenium status corresponds to dietary selenium intake and ranges from well below the RDA in eastern Europe and parts of China, to levels greatly exceeding the RDA in Venezuela, parts of the U.S., Canada and Japan (for details, see [10]). The average US male and female over 20 years of age consume 151.2 µg and 107.5 µg, respectively, of selenium from food and supplements per day [12], with both averages exceeding the RDA by more than double. High serum selenium concentrations, which reflect dietary intake, are associated with a higher occurrence of diabetes, higher fasting plasma glucose and increased glycosylated hemoglobin levels [13,14]. From these observations, it is proposed that high selenium intake could promote diabetes; however, the effect of selenium at the nutritional level is likely to be very different from the supranutritional level. Given the narrow margin between selenium deficiency, adequacy, supranutritional and toxicity, knowing the selenium status of individuals or populations will be helpful in determining the relationship between diabetes and selenium. 

A variety of factors can affect selenium status and, therefore, its function in the body, such as individual genetic variation [15,16,17], gender [18], smoking [18,19], alcoholism [20], age [18,21], disease [18,22,23] and selenium speciation [1]. The most common way to measure selenium status is using biomarkers in the plasma. Concentrations of elemental selenium, glutathione peroxidase and selenoprotein P in plasma are used as biomarkers, because they all respond to dose-dependent changes in selenium intake [24]. Selenium status can also be assessed using concentrations in toenails and hair, which are useful for monitoring long-term selenium status. According to the World Health Organization, the proposed optimal concentration of selenium in plasma for healthy adults is 39.5–197.4 ng/mL. Glutathione peroxidase reaches maximal activity when serum selenium concentrations are between 70–90 ng/mL, which can be achieved through dietary intakes of selenium 55–65 µg/day. This relationship is the scientific basis for setting the RDA in the U.S. Newer research shows that Selenoprotein P is the main supplier of selenium to tissues in the body [25,26]. Selenoprotein P reaches its maximal expression when serum selenium concentrations are considerably higher than those required to maximize glutathione peroxidase activity, around 125 ng/mL. This serum concentration is equivalent to a dietary selenium intake of 100 µg/day [25,26], which is twice the RDA, but reflective of the average selenium consumption in the U.S. Therefore, plasma selenium concentrations corresponding to optimal health are highly dynamic and based on a combination of factors that need to be considered when assessing epidemiological data, such as the findings linking serum selenium and type 2 diabetes.

## 2. Nutrition and Cancer

### 2.1. Selenium Supranutrition and Cancer Prevention

A strong body of animal and clinical evidence points to a role for selenium in counteracting tumorigenesis (for details, see [27]), particularly at supranutritional levels. Supranutritional amounts of a nutrient are levels that are above the established needs of an individual, but not toxic. In the case of selenium, it has been shown that supranutritional doses can reduce the risk of lung, prostate and colorectal cancers [28]. The possible mechanism for selenium’s chemopreventive properties is diverse, from inducing cellular senescence [29,30,31] to activating cell cycle arrest [27,32] or by intracellularly generation of ROS [33,34,35,36,37], among others. However, it cannot be said unequivocally that selenium supranutrition can prevent cancer under all circumstances [38,39]. The genetic background, baseline selenium status, age and gender of an individual are factors that influence the efficacy of selenium supplementation on chemoprevention. The type of cancer, speciation of selenium and the time of intervention dramatically vary widely amongst studies and alter the effectiveness of selenium chemoprevention, which could explain the opposite conclusions of the selenium chemoprevention studies. Furthermore, some studies show that selenium supplementation does not reduce cancer risk [40,41] and that certain selenoproteins act as cancer promoting agents [42,43]. Considering other data that link selenium and the promotion of chronic diseases [44,45,46,47], this highlights a lack of knowledge regarding the molecular mechanism of selenium functions and stresses the importance of identifying populations that may benefit from supplementation before recommending intervention. Furthermore, it is the opinion of the authors that plasma selenium concentrations, rather than selenium consumption, should be the critical factor when evaluating the relationship between selenium and chronic diseases. 

The consistent trend in human studies is that there is a U-shaped risk response to selenium intake or supplementation, especially concerning cancer. The literature has consistently shown that the greatest benefit of selenium supplementation is conferred to those with the lowest selenium status prior to selenium intervention [18,48]. Individuals with serum selenium concentrations between 120 and 160 ng/mL, a level achieved with a dietary selenium intake of 100–150 µg/day, have a reduced risk of certain cancers when compared to individuals with plasma selenium concentrations of less than 120 ng/mL [49]. Furthermore, research shows that the protective effect against cancer is reduced in individuals with a high plasma selenium status (>160 ng/mL). Currently, using these reference values, the average American consumes adequate selenium (151.2 µg/day for men and 107.5 µg/day for women) to put their plasma selenium concentrations within the range that offers the most protection against cancer. Further supplementation is beneficial for those with a low selenium status, such as people who live in selenium deficient areas; however, it may be unnecessary for most people, because selenium supplementation beyond what is necessary to reach optimal serum values offers no extra benefit and may potentially have harmful effects. 

The Nutritional Prevention of Cancer Trial (NPC) was conducted from 1983 to 1991, and 1312 individuals with a history of basal cell or squamous cell carcinomas were divided into groups and given 200 µg of selenium or a placebo. The primary end-point of the randomized control trial was measuring the incidence of basal cell and squamous cell carcinomas of the skin. Later, in 1990, the study established a second endpoint to measure all-cause mortality and total cancer mortality, total cancer incidence and the incidence of lung, colorectal and prostate cancers [28]. The study showed that 200 µg of selenium, in the form of selenium-enriched yeast containing 65%–80% selenomethionine (3–4 times above the RDA), reduced total mortality, mortality from all cancers and decreased the incidence of lung, colorectal and prostate cancer in individuals compared to those taking a placebo after a 8–10 year follow up. The participants seeing the greatest benefit were those that entered the trial with low plasma selenium concentrations (<121.6 ng/mL) [18]. In contrast, the Selenium and Vitamin E Cancer Prevention Trial (SELECT) failed to demonstrate that selenomethionine or vitamin E, alone or in combination, prevented the incidence of prostate cancer in a population of relatively healthy men [40]. 

These contradictory data may be explained by the differences in the baseline selenium levels of the participants. The participants in the NPC trial had a significantly lower selenium status at baseline than those in the SELECT trial, which suggests that those with a lower selenium status may benefit the most from selenium supplementation. The data from the SELECT trial was reviewed again [50], and it suggests that selenium benefits may have been overlooked. There was no statistically significant increased risk of prostate cancer in the group receiving vitamin E and selenium together, but there was a significantly increased risk of prostate cancer in the group that took only vitamin E. This suggests that selenium may somehow be able to blunt the increased risk of prostate cancer seen in the group taking vitamin E alone. After statistical adjustment for the marginal effects of selenium and vitamin E, the *p* value (0.02) of the interaction between the two nutrients is significant, thus indicating that selenium can reduce the risk of developing cancer. Furthermore, the NPC and SELECT trials, along with similar results from animal studies [51,52], indicate that selenomethionine is unlikely to be the best active selenium compound to counteract tumorigenesis. 

In conclusion, there is sufficient research to show that a serum selenium status of approximately 120–160 ng/mL can decrease cancer risk. Although this level is considered supranutritional, it is easily attained by most individuals in the U.S. on a regular basis without selenium supplementation. With the dramatic increase in caloric intake, regular multivitamin use and abundance of functional foods, selenium supranutritional status is likely achieved by many people without the need of further supplementation. Thus far, no benefits have been associated with serum selenium concentrations greater than 120–160 ng/mL; rather, higher levels have been linked to adverse health outcomes [11]. Therefore, people with an adequate or high selenium status are already receiving the maximized benefit from selenium and should not take additional selenium supplementation, because of the potential risks, such as the increased risk of type 2 diabetes.

### 2.2. Type 2 Diabetes and Increased Cancer Risk

Type 2 diabetes is characterized by insulin resistance and glucose intolerance and is caused by a combination of genetic and lifestyle factors, with obesity being the most common underlying factor. Even though type 2 diabetes is mostly preventable through lifestyle and diet modification [53], the prevalence of diabetes is predicted to increase following the obesity epidemic worldwide [54]. The relationship between type 2 diabetes and cancer is unclear, because there are various metabolic dysfunctions in diabetics (hyperglycemia, insulin resistance, hormonal abnormalities, *etc.*) that alter the risk of developing cancer. Other factors, such as the drugs used to manage diabetes, the diet, body composition and physical activity vary widely among individuals with type 2 diabetics, making it a disease highly variable and unique among individuals. In general, hyperinsulinemia and obesity are thought to be two main factors in type 2 diabetics that increase their risk of cancer. 

Studies show that type 2 diabetes increases the risk of several types of cancer, including liver, pancreas, colorectal, kidney, bladder, endometrial and breast and non-Hodgkin’s lymphoma [55]. The liver and pancreas are the most likely organs to develop cancer in a diabetic and often become damaged due to inflammation and oxidative stress [56], factors being hypothesized as causative factors in tumorigenesis. In addition, it is plausible that elevated insulin level could promote tumor growth by providing a nutrient-rich environment. Reproductive cancers, like breast cancer and endometrial cancers, also are increased in diabetics, even when correcting for obesity [55]. Proposed mechanisms for this include that hyperinsulinemia can decrease sex hormone binding globulin concentrations, therefore increasing bioactive forms of estrogen or stimulating androgen synthesis in ovarian stroma [57]. The increase in kidney [58] and bladder [59] cancer may be due to hyperinsulinemia, causing increased stress on these organs. Colorectal cancer is increased in type 2 diabetics [60,61]. Hyperinsulinemia, increased fecal bile acid concentrations and slower bowel transit time are thought to be the largest underlying causes of the increased cancer risk [62,63]. The cellular and humoral immune abnormalities, particularly altered neutrophil activity, in diabetics are hypothesized factors that increase the risk of non-Hodgkin’s lymphoma in type 2 diabetics [64]. Interestingly, a meta-analysis found a 16% decreased risk of prostate cancer [65] in type 2 diabetes. The basis for the decrease is thought to be due to decreased testosterone levels [66,67].

Furthermore, diabetics have increased mortality from all cancers once diagnosed. The reason for the increased mortality is unclear. Given the various metabolic disturbances present in type 2 diabetes, it is reasonable to assume that they respond to cancer treatments differently than people without the disease. For example, it is plausible that having type 2 diabetes makes chemotherapy less effective, that they are resistant to conventional treatment and that they require modified and specialized treatments. One observational study concluded that people with gastrointestinal cancers had lower blood selenium values than people without cancer; however, in most types of cancer, blood selenium values were no different from people without cancer [68]. This study suggests that selenium status is not indicative of cancer risk in type 2 diabetics and that selenium supplementation in type 2 diabetic cancer patients with low selenium levels may not increase the severity of their cancer. 

## 3. Type 2 Diabetes and Selenium

### 3.1. Linking Type 2 Diabetes and Selenium

Thirty years ago, it would seem far-fetched to associate micronutrient status and type 2 diabetes; however, epidemiological studies have shown an association between selenium and type 2 diabetes. Because the incidence of type 2 diabetes is predicted to increase dramatically in the coming decades, it should be a priority to elucidate the relationship and explain the mechanistic link between selenium and type 2 diabetes. Data from the U.S. National Health and Nutrition Examination Surveys (NHANES) indicate that serum selenium is positively correlated with an increased incidence of type 2 diabetes [13,69]. Furthermore, in the French randomized placebo-controlled Supplementation en Vitamines et Minéraux Antioxydants (SU.VI.MAX) trial, selenium was the only antioxidant nutrient tested that was positively associated with increased fasting plasma glucose, which is a precipitating factor in the development of diabetes [70]. In addition, another study indicates that individuals that had the highest baseline plasma selenium levels were at an increased risk for type 2 diabetes even when factors, such as age, sex, body mass index and smoking status were controlled [71]. 

In contrast, there are studies that demonstrate selenium protection against diabetes. One study showed that non-diabetic individuals had higher serum selenium concentrations compared to the diabetic individuals [72]. Another study reported that mean plasma selenium content in diabetic patients was significantly lower than the controls and that there was a negative correlation between plasma contents of selenium and glycosylated hemoglobin [73]. Furthermore, mean serum selenium level of diabetics has been shown to be significantly lower (64.9 ± 22.8 µg/L) than normal individuals (74.9 ± 27.3 µg/L) [74]. Another study found that 41% of people with pancreatitis and 12% of diabetics had a low selenium concentration [75]. In addition, diabetic men have lower levels of toenail selenium than the levels found among non-diabetic controls [76]. Interestingly, individuals consuming a normal diet with the highest toenail selenium levels were at the lowest risk for type 2 diabetes [77]. Women with gestational diabetes have significantly lower serum selenium concentrations than normal pregnant women [78]. In particular, another study recommends selenium supplementation for women with gestational diabetes to prevent complications [79]. 

Because a higher selenium status or intake increases the risk of type 2 diabetes and there is a growing pervasiveness of type 2 diabetes, it is imperative to examine the effect of selenium supplementation on the risk of type 2 diabetes. Interestingly, older adults with a relatively low selenium status did not show any diabetogenic effects after a six-month supplementation [80]. These results suggest that selenium in excess, not selenium itself, may be the reason for the increased risk found in those with a high selenium status and intake. This underscores the U-shaped risk response curve of selenium intake, which is further verified by the animal study that showed that both selenoprotein deficiency and a high expression of selenoprotein cause diabetogenic effects [81].

### 3.2. Proposed Mechanisms

Precise mechanistic evidence is lacking to explain the clinical and epidemiological data that selenium promotes type 2 diabetes. Thus far, in the literature, there are limited biochemical and laboratory data [82,83,84,85] that provide evidence of a mechanistic link between type 2 diabetes and selenium, with the most convincing data focusing on specific selenoproteins in glucose metabolism, but not on changes in dietary intake. One such study showed that type 2 diabetics had higher serum expression of selenoprotein P compared with normal subjects [86]. In addition, this study provided mechanistic evidence to show that selenoprotein P causes insulin resistance in hepatocytes by decreasing phosphorylation of AMPK, an important kinase in cellular homeostasis and hormone regulation. This suggests that selenium could alter the secretion profile of hepatocytes to one that favors the pro-inflammatory state associated with diabetes. However, there are animal [87,88,89,90] and case control studies [74,76,91] that suggest selenium may improve glucose metabolism. Furthermore, selenium has also been shown to have insulin-like properties [92], which qualifies it as a potential antidiabetic agent. Recently, the study that shows selenoprotein deficiency and high expression of selenoproteins both cause glucose disturbances further complicates the issue [81]. Pertinent issues are to differentiate between the effects of selenoproteins *versus* the element itself, as well as the effects of adequate selenium *versus* excess selenium. Thus, the exact relationship among long-term selenium supplementation, selenoprotein and type 2 diabetes awaits further investigation. 

Oxidative stress and low-grade chronic inflammation play a major role in the etiology, pathogenesis and complications of type 2 diabetes [93,94,95,96]. Experimental data suggest that supplementation with antioxidants, such as selenium at the nutritional level, could delay the development of type 2 diabetes by decreasing oxidative stress [97]. Many diabetic complications are thought to be caused by oxidative damage and decreased antioxidant protection. For example, studies have shown that selenium can protect against oxidative damage attributable to unregulated blood sugar [98,99]. In one animal study, hamsters fed a low selenium diet had diabetes-induced weight loss, increased plasma triglyceride and cholesterol concentrations and rapid oxidation of LDL, none of which were seen in hamsters fed a high selenium diet [100]. In addition, a single dose of streptozotocin (50 mg/kg body weight), a chemical that is toxic to pancreatic beta cells, is enough to cause significant changes in the expression of selenium-containing antioxidant enzymes in the liver of rats [101]. Furthermore, selenium’s antioxidant capacity has also been shown to help the pathology of type 2 diabetes in other ways, including preventing oxidation of low density lipoprotein [102], inhibiting pancreatic calculi [75,103], protecting against glomerular lesions [87], helping maintain normal fatty acid concentration [104], correcting expression of glucose and lactate metabolic proteins [105], preventing the loss of myofibrils and the size of myocytes [106], attenuating red blood cell damage [107], reducing malondialdehyde levels [108] and increasing the antioxidant and ultrastructure of the liver [101]. 

Both glutathione peroxidase-1 (GPX1) overexpression and knockout mice have changes in glucose metabolism, thus underscoring the necessity of oxidative signaling at a physiological level as a requirement for normal cellular functioning. Mice overexpressing GPX-1, the most abundant selenoprotein in mammals, develop hyperglycemia, hyperinsulinemia and insulin resistance by 24 weeks of age [109]. This suggests that GPX1 has a role in glucose metabolism and insulin function. Results indicated that the diabetic phenotype the mice developed was linked to decreased insulin-mediated phosphorylation of the insulin receptor and downstream kinases in both liver and muscle cells. However, a recent report showed that GPX1 overexpression mice also have increased expression of Selenoprotein S and selenoprotein T [81], suggesting that diabetic phenotypic changes may not be solely attributed to GPX1. Considering that oxidative stress precipitates insulin resistance, these results are fascinating, because insulin resistance can be developed in mice when an antioxidant protein was overexpressed. However, an increased oxidative scavenging ability does not necessarily correlate with decreased oxidative stress, and this study elegantly shows that the disruption in normal redox conditions, perhaps mediated by intracellular H_2_O_2_ signaling, can induce diabetogenic changes. Interestingly, mice that lack GPX1 are protected from a high fat diet-induced insulin resistance [110]. The authors showed that cells from GPX1 knockout mice have elevated oxidative stress compared to control cells. The GPX1 knockout mice were found to have greater glucose uptake than the control mice. Thus, the researchers concluded that the increased oxidative stress in the knockout mice activates insulin signaling, promoting insulin sensitivity and enhancing glucose uptake by cells. 

These transgenic mouse studies clearly indicate a role of selenoproteins in diabetes, but the applicability to the human epidemiological studies linking type 2 diabetes and supranutritional selenium status may be limited. One of the reasons is that most individuals participated in the clinical trials exhibit serum selenium at the adequate level to support the expression of selenoproteins. The epidemiological studies most likely are reflective of oxidative stress by body selenium deprivation or in excess. These data are extremely useful in understanding diabetes and other chronic diseases, because it helps solidify the concept that there is an optimal physiological oxidant tone [111,112,113] that is a requirement for normal metabolic function. Therefore, the negative associations between selenium and diabetes may be a direct observation of selenium excess or deprivation disturbing intracellular H_2_O_2_ signaling [114]. Over time, these disturbances could alter the oxidant tone and create a cellular environment incapable of effective insulin signaling and glucose uptake [115,116,117], ultimately manifesting in type 2 diabetes. 

## 4. Conclusions and Perspectives

**Figure 1:**
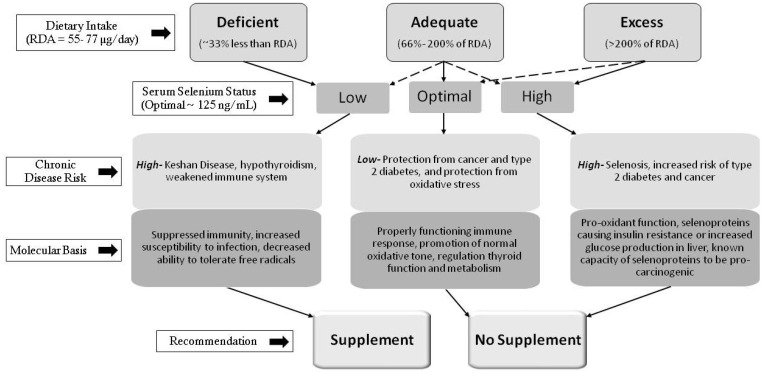
Selenium status and promotion or prevention of chronic diseases. Description of the relationship between dietary selenium and selenium status and a summary of the effects of selenium on chronic disease**.**

Currently, the amount of selenium recommended in the RDA correlates to a level that maximizes GPX1 expression. Given that the positive impacts of dietary selenium on chemoprevention occur in amounts 3–4 times greater than the RDA and that selenoprotein expression reaches a plateau at the current RDA level, the scientific basis for the determination of the RDA could be revised concerning the optimal health endpoint. Nonetheless, selenium consumption or supplementation is not as important as body selenium status in determining the potential health risks or benefits associated with an individual. The preponderance of evidence suggests that a normal or adequate selenium status is protective against both cancer and type 2 diabetes. Restoring selenium levels in individuals who are deficient in this nutrient may be protective against cancer; however, the benefit is most likely due to the elimination of deficiency, not benefits derived from a high selenium status. Selenium supplementation in individuals with a normal or high selenium status does not further reduce cancer risk and is not recommended. Furthermore, clinical and epidemiological data raise apprehension about the recommendation of selenium intake or supplementation above the RDA and its potential to promote type 2 diabetes [14,118]. The relationship between dietary selenium and selenium status, as well as the impact on human health and known mechanisms, are summarized in Figure 1.

Although these data are alarming, it is also plausible that the epidemiological data reflect increased food and caloric consumption, which is inexplicably linked to type 2 diabetes, and that elevated selenium status is merely a coincidence. At this point, due to the available epidemiological data, individuals with diabetes are advised against using any sort of selenium supplementation [45,69]. One exception could be for those who live on land deficient in selenium, including northwestern, northeastern and the southeast U.S., New Zealand and the “selenium-deficient belt” spreading across northeastern China into the southwestern provinces. 

Assessed from a nutrigenomic perspective, for some individuals, selenium supplementation might outweigh the risks. If people are able to identify particular genes that predispose them to one chronic condition, such as cancer or type 2 diabetes, they could theoretically personalize a nutrition or diet therapy for optimal selenium intake. In particular, populations and individuals with genetic variations due to genetic polymorphisms in selenoprotein genes would also benefit from a personalized approach to evaluate their selenium intake. Lastly, when people are deciding whether selenium supplementation would work for them, they should consider the stage of their disease and the impacts of potential side effects. Researchers should employ a nutrigenomics-driven approach to clarify the relationship between selenoprotein function and selenium status. 
